# Electroacupuncture Alleviates Postoperative Cognitive Dysfunction in Aged Rats by Inhibiting Hippocampal Neuroinflammation Activated via Microglia/TLRs Pathway

**DOI:** 10.1155/2017/6421260

**Published:** 2017-06-08

**Authors:** Pei-pei Feng, Pu Deng, Li-hua Liu, Qi Ai, Jie Yin, Zhe Liu, Gai-mei Wang

**Affiliations:** ^1^Department of Neurobiology & Acupuncture Research, The Third Clinical College, Zhejiang Chinese Medical University, Hangzhou 310053, China; ^2^Medical Department for Senior Cadres, The Third Affiliated Hospital of Zhejiang Chinese Medical University, Hangzhou 310005, China

## Abstract

Neuroinflammation has been suggested to be involved in the pathogenesis of postoperative cognitive dysfunction (POCD). Electroacupuncture (EA) is an irreplaceable method in traditional Chinese medicine that is used for treating neurodegenerative diseases in clinical and experimental studies. The aim of this study was to examine whether EA improves cognitive dysfunction caused by surgery and to investigate the pathological mechanism of TLR2 and TLR4 in the hippocampus of aged rats. A rat model of POCD was established and treated with EA or minocycline. Both EA- and minocycline-treated rats performed significantly better than untreated operated rats in spatial memory tasks of the Morris water maze (MWM) test, spending comparatively greater amounts of time in the target zone during the probe test. Additionally, decreased levels of proinflammatory cytokines (IL-1*β*, IL-6, TNF-*α*, and HMGB1) and decreased TLR2 and TLR4 protein expression in the hippocampus of EA- and minocycline-treated rats were detected. Our data suggested that EA treatment alleviated the cognition performance deficit and neuroinflammation in aged rats following surgery, which may be mediated by inhibiting the expression of hippocampal neuroinflammatory cytokines through the microglia/TLR2/4 pathway.

## 1. Introduction

Cognitive impairment after anesthesia and surgery (postoperative cognitive dysfunction (POCD)) is a recognized clinical phenomenon that was first described in older persons following surgery by Bedford in the Lancet in 1955 [1]. POCD is a common complication that refers to a decline in cognitive function following surgery, which is characterized by an impairment of memory, concentration and comprehension, and a decreased ability to process information [2, 3]. It is also associated with significant morbidity and mortality in elderly patients and adversely affects quality of life and social dependence [4]. POCD is present in 25.8% of patients aged at least 60 years 1 week after surgery and in 9.9% of patients 3 months after a major noncardiac surgery [5]. Aging and surgery are specific risk factors for the occurrence and development of POCD [6].

Neuroinflammation has been suggested to be involved in the pathogenesis of POCD [7–9], although the exact mechanism is not clear [10, 11]. Activated microglia, the immune cells of the brain, and their secreted factors are key mediators of neuroinflammation in the hippocampus and may contribute to cognitive dysfunction [12]. Anesthetics and surgical trauma lead to neuroinflammation and cognitive dysfunction by upregulating the release of proinflammatory cytokines, such as tumor necrosis factor-*α* (TNF-*α*), interleukin- (IL-) 1*β*, and IL-6, and by activating microglia in the hippocampus [13, 14]. Microglia, in normal aging, are associated with a “sensitized” or “primed” phenotype [15], which appears to be the source of an amplified neuroinflammation response and exacerbated cognitive impairment [6, 14]. Excessive production of cytokines by primed microglia, however, may cause long-lasting behavioral and cognitive complications in aged rats [16]. High-mobility group box-1 (HMGB1) is a nuclear protein that, upon its extracellular release, acts as a cytokine and is involved in various inflammatory processes [17, 18]. HMGB1 can interact with Toll-like receptor-2 and -4 (TLR2/4) to mediate the release of proinflammatory cytokines and induce memory abnormalities in the brain [17]. Several of these mediators have been shown to influence inflammatory processes in the brain, leading to the activation of microglia and concurrent endogenous production of proinflammatory cytokines [9, 19]. TLR4 is highly expressed in brain microglia following surgery, and excessive inflammation resulting from activation of the HMGB1/TLR4 pathway in the brain has been implicated in neurodegenerative pathologies [20, 21]. Blocking HMGB1 signaling can downregulate the expression of inflammatory pathway genes in aged rat brains [22].

Minocycline is a tetracycline derivative that was considered to be an effective protective agent against neurodegenerative disease in various experimental models [23]. Because of its high tolerance and excellent penetration into the brain, minocycline has been clinically tested for some neurodegenerative diseases, acting via multiple mechanisms, including anti-inflammatory effects [24]. The anti-inflammatory properties of minocycline include the reduction of proinflammatory cytokines and suppression of microglial activation, which is involved in some signaling pathways [24, 25]. In addition, minocycline has been well established to exert neuroprotective effects via inhibition of TLR4-microglia pathway in various experimental animal models [26, 27]. Therefore, minocycline was used as a positive control in the present study.

Electroacupuncture (EA), a commonly used acupuncture method performed by applying a pulsating electrical current to acupuncture needles, is an effective therapeutic procedure for treating cognitive impairment and has been widely used for decades in clinical and experimental research for stroke, Alzheimer's disease (AD), and vascular dementia (VD) [28–30]. Accumulating evidence has demonstrated that the therapeutic effect of EA may be related to the inhibition of neuroinflammation [31, 32]. However, the effects of EA on cognitive impairment in POCD have not been studied. Thus, we tested the hypothesis that EA treatment can improve postoperative cognitive dysfunction and the underlying mechanisms may be related to inhibition of hippocampal neuroinflammation via the microglia/TLRs pathway in aged rats that underwent abdominal surgery.

## 2. Materials and Methods

### 2.1. Animals and Groups

Eighty Sprague-Dawley male rats (21–23 months old, weighing 500–600 g) were purchased from the Experimental Animal Center of Zhejiang Chinese Medical University (Zhejiang, China) and were housed in groups of five in plastic cages with soft bedding at the University Animal Care facility, with an artificial 12/12 hour light-dark cycle. Animals received food and water ad libitum with a constant room temperature of 23–25°C and a relative humidity of 40–70%.

All animal procedures performed in this work followed guidelines in accordance with the Regulations for the Administration of Affairs Concerning Experimental Animals and were approved by the Animal Care and Welfare Committee of Zhejiang Chinese Medical University, Zhejiang, China. Rats were randomly assigned to four groups. In the control group (*n* = 20), rats did not undergo surgery but were injected with sterile saline to control for the effects of stress. The remaining 60 rats were divided into 3 parallel operation groups (surgery control, surgery + drug and surgery + EA;* n* = 20, resp.). All rats were trained in the Morris water maze (MWM) for 5 days. Subsequently, rats in the operation groups underwent partial hepatectomy under isoflurane anesthesia, and all rats were tested by the MWM again on postoperative days 3 and 7. Briefly, the liver was exposed through a 1-2 cm midline abdominal incision. The left lateral lobes of the liver (corresponding to approximately 30% of the organ) were excised. The wound was then infiltrated with 0.25% bupivacaine for postoperative analgesia and closed using a sterile suture [14].

### 2.2. EA and Drug Treatment

Acupuncture was performed at the acupoints of Baihui (GV 20) and Dazhui (GV 14) for rats in the surgery + EA group each day during the experimental procedure. Sterilized disposable stainless steel needles with a 0.3 mm diameter were inserted as deep as 3–5 mm. The GV 20 acupoint is located above the apex auriculate on the midline of the head. The GV 14 acupoint is located on the posterior midline and in the depression below the spinous process of the 7th cervical vertebra in the prone position. The ends of the needles were attached to a pair of electrodes from an electrical stimulator (HANS 200E, Huawei Co. Ltd., Beijing, China). The EA parameters were set as follows [33]: a constant square wave current output (15 Hz), with the intensities remaining at 1 mA (causing the slight vibration of muscles around acupoints) for 30 minutes once a day starting from day 1 postoperatively. Animals were awake and calmed by placing their heads in black hoods with no physical restraint during EA treatment. The same calming procedure was performed for rat in the control, surgery control, and surgery + drug groups. Rats in the surgery + drug group received minocycline by intraperitoneal injection (45 mg/kg/d) [26, 34].

### 2.3. Behavioral Testing

The MWM is a hippocampal-dependent test of spatial learning and memory for rodents [35]. Rats were released into the water facing the wall of the pool from one of four randomly assigned release points (N, W, S, and E). Animals were allowed 60 sec per trial to find the platform (diameter 10 cm) that was placed 2 cm beneath the water surface in the center of one quadrant. Between each of the four trials, animals were allowed to sit on the platform for 10 sec. If an animal did not find the platform within the allowed time, an experimenter would guide the animal to the platform, and animals were allowed to rest on the platform for 30 sec. Rats received four trials per day for 5 consecutive days and were removed if a rat failed to find the platform in the 5th training days. Animals underwent surgery on the sixth day. On postoperative days 3 and 7, rats were subjected to a spatial probe test in which the platform was removed from the pool. The spatial probe test reveals whether animals can maintain their initial learning of the platform's position. The swimming latency to the platform was recorded by a video tracking apparatus mounted on the ceiling, and digital images were analyzed by water maze software (SMART v3.0.02 Image, Spain).

### 2.4. Tissue Treatment

Animals were sacrificed following anesthesia with sodium pentobarbital, and the brains were rapidly removed and frozen on ice. Hippocampal tissues of 5 rats in each group were quickly separated and stored in liquid nitrogen prior to use. The remaining animals in each group were perfused with cold physiological saline, followed by a cold 4% paraformaldehyde solution in 0.1 M PBS (pH 7.4). Next, the brain tissue was postfixed in 4% paraformaldehyde in PBS for 24 hours, followed by dehydration in 15% sucrose for 1 day and in 30% sucrose for up to 3 days; the brain tissue was subsequently embedded in an optimum cutting temperature (OCT) tissue freezing medium and sectioned for immunofluorescence.

### 2.5. Enzyme-Linked Immunosorbent Assay (ELISA)

Snap-frozen hippocampus samples were homogenized in lysis buffer (Applygen, Beijing, China) containing a protease inhibitor cocktail. The samples were then centrifuged at 12,000*g* at 4°C for 15 min. The levels of IL-1*β*, IL-6, TNF-*α*, and HMGB1 were measured in the supernatants, which were diluted before measurement in order for the cytokine levels to be within the linear portion of the sigmoid curve, as determined by a Parameter™ IL-1*β*, IL-6, TNF-*α*, and HMGB1 Immunoassay kit (R&D Systems, USA), according to the manufacturer's instructions. The intensity of the color was measured at a wavelength of 450 nm with a SpectraMax M4 microplate reader (MolecularDevices, USA). Each sample was examined in duplicate and averaged for data analysis. Five rats in each group were randomly selected for ELISA.

### 2.6. Protein Extraction and Western Blotting

The hippocampus tissue samples were homogenized with lysis buffer containing a cocktail of phosphatase and proteinase inhibitors and PMSF (Beyotime, Shanghai, China). Following denaturation, the lysates were separated on a 10% SDS-PAGE gel and transferred to polyvinylidene difluoride PVDF membranes (Bio-Rad, Hercules, CA, USA). The membranes were blocked with 5% nonfat powdered milk in TBST (Tris-buffered saline containing 0.1% Tween 20) for 1 h at room temperature (RT) and then incubated overnight at 4°C with a monoclonal rabbit anti-TLR4 (1 : 1000, Proteintech Group), anti-TLR2 (1 : 1000, Proteintech Group), or anti-GAPDH (1 : 5000, Proteintech Group) primary antibody. After washing in TBST, the membrane was incubated for 1 h at RT with a HRP-conjugated goat anti-rabbit antibody (1 : 10000; Jackson ImmunoResearch Laboratories, West Grove, PA, USA), and the protein bands were visualized using a Immun-StarTM HRP Chemiluminescence Kit (Bio-Rad). Images of the bands were recorded using the ImageQuant LAS 4000 system (GE Healthcare, Hino, Japan), and the band intensities were quantified using ImageQuant TL software (version 7.0, GE Healthcare). The amount of proteins was quantified (Quantity One, BioRad) and reported relatively to GAPDH. Three rats in each group were randomly selected for western blotting.

### 2.7. Double-Immunofluorescence Labeling Procedures

Coronal brain slices were cut at a 16 *μ*m thickness on a cryostat microtome (Microm HM 550; Thermo). Sections at the level from −2.3 mm to −4.16 mm from the bregma were chosen by preliminary experiments. Sections were blocked with 5% BSA at 37°C for 1.5 h and then incubated overnight at 4°C with an anti-CD11b antibody (mouse monoclonal, 1 : 200; AbD, USA) along with either an anti-TLR4 antibody (rabbit polyclonal, 1 : 100; Santa Cruz, sc-30002) or an anti-TLR2 antibody (goat polyclonal, 1 : 50; Santa Cruz, sc-16237). Following washing with PBS, the sections were incubated with an Alexa Fluor 647-conjugated goat anti-mouse secondary antibody (1 : 200, Jackson ImmunoResearch Laboratories, West Grove, PA, USA), an Alexa Fluor 488-conjugated goat anti-rabbit secondary antibody (1 : 100; Jackson ImmunoLabs), and an Alexa Fluor 488-conjugated donkey anti-goat secondary antibody (1 : 100; Jackson ImmunoLabs) at 37°C for 1 h. Finally, the slides were examined using a Nikon A1R confocal laser-scanning microscope, and positive cells in the hippocampal CA1, CA3, and DG region were captured using NIS elements AR software.

### 2.8. Statistical Analysis

All data were expressed as the means ± standard deviation (SD). Data from ELISA, western blot analysis, and the spatial probe test were analyzed using one-way ANOVA and the Bonferroni multiple comparison test. One-way repeated measures ANOVA was used to analyze the training behavioral parameters. A value of* P* < 0.05 was considered statistically significant.

## 3. Results

### 3.1. EA Improves the Spatial Memory of Rats following Surgery

**Figure 1:**
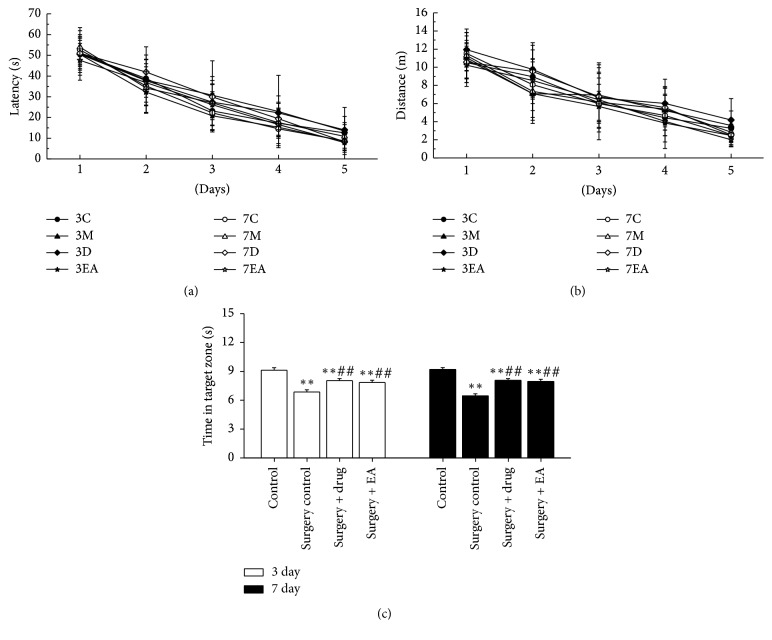
Swim tests of rats for 5 consecutive training days and the spatial probe test in the Morris water maze. (a) Distance to target across training days, (b) latency stage to target across training days, and (c) swim time in target zone during the spatial probe test. The results are presented as the mean ± SD. All of the results were compared at the same time point. ^*∗∗*^*P* < 0.01 versus the control group; ^##^*P* < 0.01 versus the surgery control group. C, M, D, and EA: control, surgery control, surgery + drug, and surgery + EA group. 3 day and 7 day: operation group on day 3 and day 7 postoperatively, respectively.

To evaluate the effects of EA on hippocampal-dependent spatial learning and memory, a MWM was constructed. The place navigation test took was performed before surgery. Repeated measures ANOVA of swim data revealed no significant effects of time (day) on both latency (Figure 1(a),* P *> 0.05) and distance (Figure 1(b),* P *> 0.05) among all groups. The result indicated that all rats showed the same improvement in spatial learning and memory over time. Next, the spatial probe test was conducted postoperatively. Analysis of the time in the target zone (Figure 1(c)) during the spatial probe test of rats in the operation groups (surgery control, surgery + drug, and surgery + EA) revealed a significant impairment compared to the control group (*P *< 0.01), which was observed on postoperative day 3 (*P *< 0.01) and day 7 (*P *< 0.01). In the surgery + drug and surgery + EA groups, the impairments were improved on day 3 (*P *< 0.01) and day 7 (*P *< 0.01) compared to rats in the surgery control group at the same time points. These results demonstrate that surgery impaired spatial learning and memory and that both EA and drug improved the sickness response in aged rats.

### 3.2. EA Reduces the Level of the Proinflammatory Cytokines IL-1*β*, IL-6, TNF-*α*, and HGMB1 in the Hippocampus following Surgery

**Figure 2:**
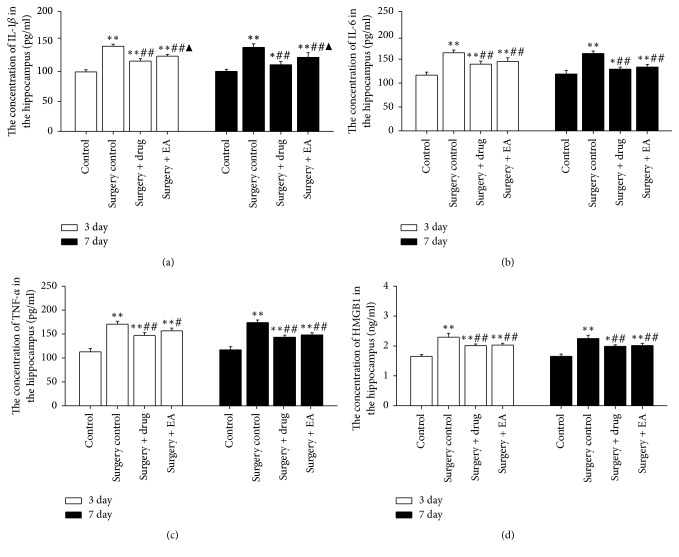
Effects of EA treatment on the levels of IL-1*β*, IL-6, TNF-*α*, and HMGB1 in the hippocampus. The hippocampal concentrations of IL-1*β* (a), IL-6 (b), TNF-*α* (c), and HMGB1 (d) of rats were measured on day 3 and day 7 postoperatively. The bars represent the mean ± SD. Five rats were included in each group. All of the results were compared at the same time point. ^*∗*^*P* < 0.05; ^*∗∗*^*P* < 0.01 versus the control group; ^##^*P* < 0.01 versus the surgery control group; ^▲^*P* < 0.05 versus the surgery + drug group. 3 day and 7 day: operation group on day 3 and day 7 postoperatively, respectively.

To investigate whether EA can alter the expression of proinflammatory cytokines in the hippocampus, IL-1*β*, IL-6, TNF-*α*, and HGMB1 were measured in tissue samples. Data analysis of ELISA revealed that the expression levels of IL-1*β*, IL-6, TNF-*α*, and HGMB1 in the operation groups were increased on postoperative day 3 (*P *< 0.01) and day 7 (*P *< 0.01) compared to the control group. The expression levels of IL-1*β*, IL-6, TNF-*α*, and HGMB1 were decreased on postoperative day 3 (*P *< 0.01,* P *< 0.01, resp.) and day 7 (*P *< 0.01,* P *< 0.01, resp.) in the surgery + drug and surgery + EA groups compared to the surgery control group at the same time points (Figures 2(a)–2(d)). No significant differences were observed for IL-6, TNF-*α*, and HMGB1 expression on day 3 and day 7 postoperatively between the surgery + drug and surgery + EA groups (*P* > 0.05) (Figures 2(b), 2(c), and 2(d)). In sum, EA downregulated the expression of IL-1*β*, IL-6, TNF-*α*, and HGMB1 from day 3 to day 7 postoperatively, indicating that EA could improve the cognitive function by decreasing the levels of these inflammatory cytokines in the hippocampus. Moreover, the therapeutic effect of EA is similar to minocycline in the present study (Figures 2(a)–2(d)).

### 3.3. EA Decreases the Expression of TLR 4/2 in the Hippocampus following Surgery

**Figure 3:**
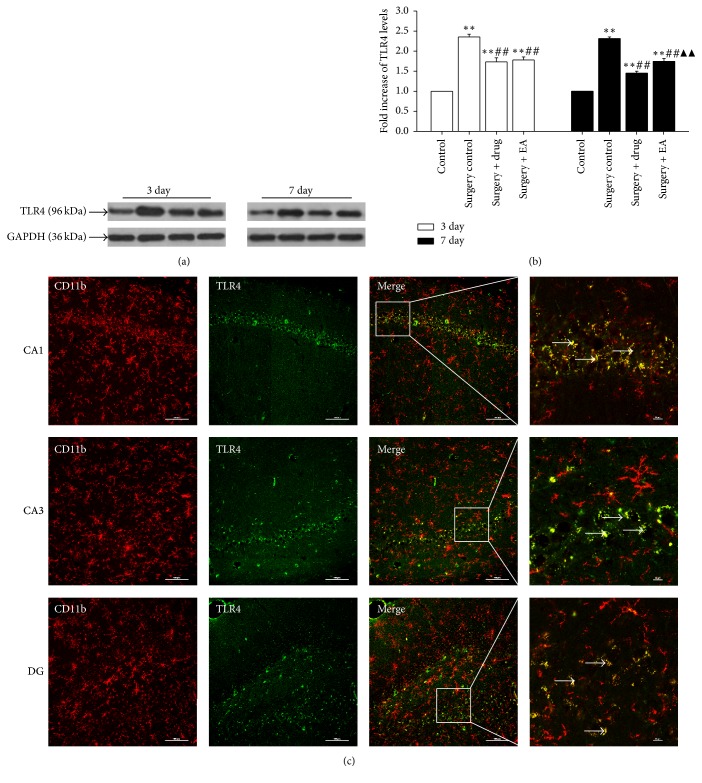
Effects of EA treatment on the levels of TLR4 in the hippocampus. (a) Changes in the expression of TLR4 protein levels in the hippocampus at different time points in the control, surgery control, surgery + drug, and surgery + EA groups. (b) The bars represent the mean ± SD. Three rats were included in each group. All of the results were compared at the same time point. ^*∗∗*^*P* < 0.01 versus the control group; ^##^*P* < 0.01 versus the surgery control group; ^▲▲^*P* < 0.01 versus the surgery + drug group. (c) Double-immunofluorescence staining of CD11b (red) together with TLR4 (green) in the hippocampal CA1, CA3, and DG region on postoperatively day 3 in the surgery control group. Scale bar for vertical panels 3 and 4 = 100 *μ*m and 10 *μ*m, respectively.

**Figure 4:**
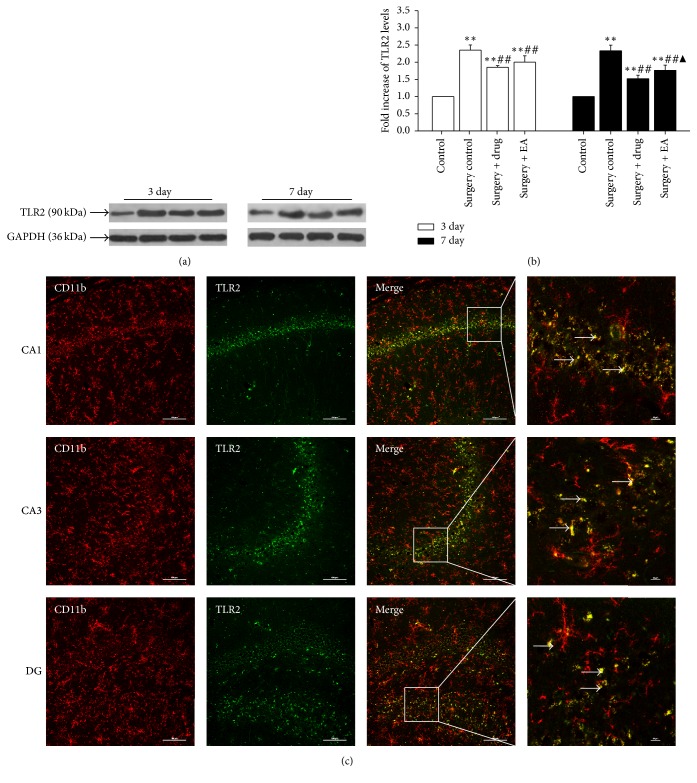
Effects of EA treatment on the levels of TLR2 in the hippocampus. (a) Changes in the expression of the TLR2 protein levels in the hippocampus at different time points in the control, surgery control, surgery + drug, and surgery + EA groups. (b) Bars represent the mean ± SD. Three rats were included in each group. (c) Double-immunofluorescence staining of CD11b (red) together with TLR2 (green) in the hippocampal CA1, CA3, and DG region on postoperatively day 3 in the surgery control group. All of the results were compared at the same time point. ^*∗∗*^*P* < 0.01 versus the control group; ^##^*P* < 0.01 versus the surgery control group; ^▲^*P* < 0.05 versus the surgery + drug group. Scale bar for vertical panels 3 and 4 = 100 *μ*m and 10 *μ*m, respectively.

As demonstrated in Figures 3 and 4, on postoperative day 3 and day 7, the expression levels of TLR4 (Figure 3) and TLR2 (Figure 4) in the operation groups (surgery control, surgery + drug, and surgery + EA) were significantly increased compared to those in the control group at the same time point (*P *< 0.01). Subsequently, the expression levels of TLR4 and TLR2 were significantly decreased over time following drug and EA treatment (*P *< 0.01). The results of the immunofluorescence experiments (Figures 3(c) and 4(c)) demonstrated a significantly stronger expression of TLR4 and TLR2 in the hippocampal CA1, CA3, and DG regions. Based on these data, we deduced that the expression of TLR4 and TLR2 changed with the intensity of neuroinflammation and activation of microglia, suggesting that EA treatment may improve cognitive function following POCD via suppressing TLR4 and TLR2 expression.

## 4. Discussion

In the present study, our data showed that EA treatment can improved the spatial memory of rats postoperatively. The potential effective mechanism of EA may be related to decreased microglial activation and downregulation of IL-1*β*, IL-6, TNF-*α*, and HMGB1 via the microglia/TLRs pathway. Activation of the above-mentioned pathway results in hippocampus-dependent memory impairments in aged rats.

In this study, we present inspiring results: the spatial memory of aged rats following surgery can be improved with EA by stimulating the “Baihui” and “Dazhui” points. The MWM was designed as a method to assess spatially or place learning in rodents [36]. We observed spatial learning and memory in rats for 5 consecutive days [37], the results of which showed no significant difference preoperatively in the various group of animals, suggesting that all rats had the same learning and memory capacity before surgery. The spatial probe test is a common detection method that reveals whether animals can maintain their initial learning of the platform's position. In the present study, the outcome of the spatial probe test demonstrates that animals in the surgery + EA group and the surgery + drug group spent similarly longer amounts of time in the target zone than animals in the surgery control group that, in turn, rats in the operation groups spent a shorter time compared to rats of the control group. Therefore, EA provided a positive effect on the learning of aged rats postoperatively, and we therefore propose that EA treatment may be beneficial for cognitive function by reducing neuroinflammation in aged rats following surgery.

Neuroinflammation, especially hippocampal neuroinflammation, is one of the important mechanisms in POCD pathogenesis for cognitive decline and dementia [8, 9, 38]. Minocycline is an anti-inflammatory drug that has been well established to exert neuroprotective effects in various experimental animal models of neurodegenerative diseases [26]. In addition, EA may improve cognitive decline in clinical [39, 40] and animal studies [29]. To confirm EA's neuroprotective effect, some classical inflammatory cytokines were detected in this study. Significantly, elevated levels of proinflammatory cytokines, such as IL-1*β*, IL-6, and TNF-*α*, in key brain regions responsible for mediating memory formation, such as the hippocampus, have been detected. These cytokines have been shown to impair memory in young adult rats [41] and can exacerbate the proinflammatory profile of aged rats [14, 19]. IL-1*β* is central to the inflammatory response and is a key mediator and modulator of an array of associated biological functions [42]. In addition to a direct effect of the proinflammatory cytokines, such as IL-1*β* [43] and IL-6, on neuronal functions that are essential for learning and memory, inflammatory factors may also indirectly influence neuronal functioning [44]. TNF-*α* is a multifunctional proinflammatory cytokine that is predominantly secreted by macrophages and microglia [45]. Inhibition of TNF-*α* led to inhibition of the activation of NF-*κ*B signaling, which in turn inhibited the production of key proinflammatory cytokines, including TNF-*α*, IL-1*β*, and IL-6, in the hippocampal tissues of the aged rats [46]. HMGB1 is an abundant, evolutionarily conserved nonhistone protein and has a surprising additional function as a secreted protein with a central role in inflammation caused by tissue damage [47]. Extracellular HMGB1 binds to cell surface TLRs to initiate signaling that culminates in the expression of inflammatory cytokines, such as IL-6 and IL-1*β* [48]. On the other hand, proinflammatory cytokines may active the microglia. Thus, a vicious circle may be created that may exacerbate the impairment of cognitive function. Recent studies have shown that IL-1*β*, IL-6, TNF-*α*, and HMGB1 played an important role in POCD pathogenesis [17, 46, 49]. Hence, we chose to explore all of their effects and involvement in EA treatment for cognitive decline postoperatively. We observed that EA treatment could significantly suppress the expression of IL-1*β*, IL-6, TNF-*α*, and HMGB1 in the hippocampus after surgery. More importantly, these effects of EA were similar to those observed in minocycline-treated rats. Therefore, EA can ameliorate cognition decline that is possibly caused by hippocampal neuroinflammation in aged rats that have undergone surgery [50].

TLRs play an important role in the innate immune response, and emerging evidence indicates their role in brain injury and neurodegeneration [51]. TLRs are pattern recognition receptor that participates in the inflammatory response by producing numerous proinflammatory factors via various pathways. Activation of the TLR pathway increases the expression of various proinflammatory cytokines, such as IL-6, IL-1, and TNF-*α* [52, 53]. HMGB1 is a classic danger-associated molecular pattern and plays a pivotal role in mediating the acute damage response and subsequent inflammatory processes [54]. It can interact with multiple receptors, including RAGE, TLR2, and TLR4, to mediate the release of proinflammatory cytokines in microglia [48, 55], which constitutes the predominant innate immune cells in the brain and mediate responses to pathogens and injury [9]. It has been demonstrated that brain injury and neurodegeneration can activate the expression and release of HMGB1 in damaged neuron of the brain, which in turn are involved in the release of neuroinflammatory cytokine via the innate immune receptors TLR4 and TLR2 in microglia [51, 56, 57]. It has also been demonstrated that TLR-induced activation of microglia and the release of proinflammatory molecules are responsible for neurotoxic processes in various CNS diseases [58]. The present study explored the role of these receptors in the progression of POCD pathologies and the possible role of EA on TLR4/2 expression. Our results from western blotting analyses and double-immunofluorescence demonstrated that surgery stimulates TLR4/2 expression in the hippocampal microglia and that EA treatment can decrease the expression of TLR4/2 in the hippocampus after surgery to an extent similar to that of minocycline. This finding is consistent with a recent report that TLR4 expression levels were lower in rats treated with minocycline [27]. Therefore, the increased expression of TLR4/2 plays a destructive role in the cognition of aged rats, and treatment with EA may alleviate POCD by inhibiting hippocampal neuroinflammation activated via the microglia/TLR4/2 pathway in aged rat following surgery. Based on the above-mentioned experimental results, we can conclude that the effect of EA on POCD is similar to that of minocycline. In addition, EA treatment has multiple targets. Therefore, we speculated that the combination of EA and minocycline may play a more prominent role in the treatment on POCD. However, this needs to be confirmed through subsequent experiments.

## 5. Conclusions

In conclusion, EA can alleviate POCD in aged rat via inhibition of the hippocampal microglia activation and subsequent neuroinflammation through the microglia/TLR4/2 signaling pathway. EA treatment may be of therapeutic value for POCD, limiting the deleterious impact of excessive neuroinflammation in the initiation and development of POCD pathologies.
